# Licoisoflavone B alleviates psoriasis via SCD1-targeted lipid metabolism reprogramming and suppression of Th17/IL-17–mediated inflammation

**DOI:** 10.3389/fphar.2026.1754729

**Published:** 2026-02-16

**Authors:** Yao Liu, Vincent Kam Wai Wong, Jiao Liu, Manling Jiang, Chenxu Yang, Xiang He, Junyi Wang, Lei Zhang, Anying Xiong, Qin Ran, Xiaolan Li, Keyue Wang, Mufan Li, Peng Song, Liang Jin, Guoping Li

**Affiliations:** 1 Dr. Neher’s Biophysics Laboratory for Innovative Drug Discovery, State Key Laboratory of Mechanism and Quality of Chinese Medicine, Faculty of Chinese Medicine, Macau University of Science and Technology, Macao, China; 2 Dr. Neher’s Biophysics Laboratory for Innovative Drug Discovery, State Key Laboratory of Mechanism and Quality of Chinese Medicine, Macau University of Science and Technology, Macau, China; 3 Macau University of Science and Technology Zhuhai MUST Science and Technology Research Institute, Macao, China; 4 Laboratory of Allergy and Precision Medicine, Department of Respiratory Medicine, Chengdu Institute of Respiratory Health, Affiliated Hospital of Southwest Jiaotong University, The Third People’s Hospital of Chengdu, Chengdu, China; 5 College of Medicine, Southwest Jiaotong University, Chengdu, China

**Keywords:** Licoisoflavone B, lipidmetabolism, psoriasis, skin, stearoyl-CoA desaturase 1, traditional Chinese medicine

## Abstract

**Introduction:**

Psoriasis is a chronic inflammatory skin disorder driven by dysregulated immune responses, Th17 cells activation, and keratinocytes hyperproliferation. Despite advances in therapies, high costs and adverse effects limit their utility. Licoisoflavone B (Lico B), bioactive flavonoid derived from licorice, exhibits anti-inflammatory and metabolic modulating properties, yet its mechanisms in psoriasis remain unexplored.

**Methods:**

We employed integrative bioinformatics, including target prediction, differential expression analysis, and weighted gene co-expression network analysis to identify psoriasis-associated hub genes linked to Lico B. Functional enrichment was analyzed via GO and KEGG pathway. Molecular docking evaluated Lico B’s binding affinity to candidate target. The effects of Lico B on Stearoyl-CoA Desaturase 1 (SCD1) expression, lipid metabolism, IL-17–induced keratinocyte proliferation, and Th17 differentiation.

**Results:**

Bioinformatics revealed Lico B’s targets were enriched in lipid metabolism and cell cycle pathways. SCD1 emerged as a key target, supported by strong binding affinity in docking studies. Experimentally, Lico B attenuated IL-17–induced SCD1 upregulation and lipid droplet accumulation in keratinocytes. It suppressed hyperproliferation markers (KRT17/Ki67) in cells and imiquimod-induced psoriatic mice. Furthermore, Lico B reduced Th17 differentiation and IL-17 production in murine models, demonstrating dual antiproliferative and immunomodulatory effects.

**Conclusion:**

Lico B alleviates psoriasis by targeting SCD1 to modulate lipid metabolism, inhibit keratinocyte hyperproliferation, and dampen Th17/IL-17–driven inflammation. This multimodal mechanism positions Lico B as a novel therapeutic candidate for psoriasis and related inflammatory-metabolic dermatoses.

## 1 Introduction

Psoriasis is a chronic, immune-driven skin disease characterized by keratinocytes (KCs) hyperproliferation and persistent inflammatory immune cell infiltration (Grayson, 2012; Armstrong and Read, 2020). Patients with psoriasis manifesting as persistent skin lesions, recurrent flare-ups, and considerable pruritus. Substantial progress has been achieved in the treatment of psoriasis through phototherapy, topical and systemic medications, and biologic therapies (Chan, 2025; Gelfand et al., 2024; Prema and Shanmugamprema, 2025). However, these therapeutic options are undermined by several factors that impair patients’ quality of life, such as the high cost of biologics, uncertainty in long-term efficacy, severe adverse reactions, and frequent relapse after treatment withdrawal (Guo et al., 2023; Liu et al., 2024). Therefore, it is imperative to explore new and effective therapeutic targets to achieve precise treatment and alleviate the symptoms of psoriasis.

Dysregulated lipid metabolism plays a pivotal role in promoting cutaneous inflammation and keratinocyte hyperproliferation, which is recognized as a key contributor to the pathogenesis and progression of psoriasis (Seiringer et al., 2024; Kanno et al., 2023; Zhang X. et al, 2022). Emerging clinical evidence demonstrated a strong correlation between fatty acid composition and disease severity, with the higher Psoriasis Area and Severity Index (PASI) scores showing a significant association with low circulating levels of omega-3 (n-3) polyunsaturated fatty acids (PUFAs), particularly docosahexaenoic acid (DHA) (Wang et al., 2023; Myśliwiec et al., 2017). Imbalances in lipid profiles not only promote excessive cytokine production by immune cells, exacerbating cutaneous inflammation, but also directly drive keratinocyte proliferation, leading to epidermal thickening (Cai et al., 2025; Trompette et al., 2022; Shan et al., 2024). For example, an altered ratio of oleic acid to linoleic acid has been shown to induce keratinocyte hyperproliferation (Wang et al., 2023). Therefore, restoring lipid metabolic homeostasis may alleviate skin inflammation and epidermal hyperplasia, ultimately improving psoriatic lesions. Among lipid-metabolizing enzymes, stearoyl-CoA desaturase 1 (SCD1) serves as the rate-limiting enzyme in fatty acid desaturation and plays a central role in maintaining lipid homeostasis (Liu et al., 2011). Dysregulated SCD1 activity disrupts monounsaturated fatty acid production, promotes lipid droplet accumulation, and enhances cellular proliferative capacity (Zhang Y. et al, 2022). However, its functional contribution to psoriatic skin inflammation and keratinocyte hyperproliferation remains unclear.

Traditional Chinese medicine (TCM) represents one of the oldest therapeutic systems in use worldwide. Bioactive compounds extracted from this TCM have attracted increasing interest as potential therapeutic candidates (Hsu et al., 2014; Wang et al., 2021). Licorice, a widely used traditional herbal medicine, exhibits potent antioxidant, anti-inflammatory, antibacterial, and antiviral properties, and has shown therapeutic potential in the management of psoriasis. However, the active components and therapeutic targets mediating their effects remain unclear, thereby limiting its clinical application (Wei et al., 2024; Deng et al., 2024). Lico B, a bioactive isoflavone component derived from licorice, has been shown to exhibit multiple pharmacological activities including hepatoprotection (Wu et al., 2024), and antitumor, antiviral, and antibacterial actions (Faisal Ahmed et al., 2025; Liu et al., 2019; Fukai et al., 2002). Recently, studies highlighted the anti-inflammatory and antioxidant effects of Lico B (Wu et al., 2024; Toda and Shirataki, 2001). Specifically, Lico B has been demonstrated to attenuate mitochondrial damage and hepatic inflammation induced by acetaminophen by enhancing mitophagy (Wu et al., 2024). However, whether Lico B can suppress keratinocyte proliferation and thereby alleviate epidermal hyperplasia in psoriasis remains unknown.

Bioinformatics analysis has become an essential approach for identifying aberrantly expressed genes and potential therapeutic targets (Gauthier et al., 2019; Akalin, 2006). Among these methods, weighted gene co-expression network analysis (WGCNA) is a widely applied systems biology tool that delineates gene modules with highly correlated expression patterns in transcriptomic datasets, thereby enabling the discovery of disease-related biomarkers and candidate targets (Langfelder and Horvath, 2008). Once combined with target prediction and molecular docking, these integrative analyses enable the precise identification of bioactive targets of traditional medicinal compounds (Wu et al., 2022; Zhao et al., 2025).

In our study, we combined integrative bioinformatics analysis with *in vitro* and *in vivo* validation to investigate the anti-psoriatic mechanisms of Lico B. We demonstrated that Lico B ameliorates psoriatic epidermal hyperplasia while targeting the lipid metabolic enzyme SCD1, as evidenced by the suppression IL-17-induced SCD1 upregulation and lipid droplet accumulation in keratinocytes. In addition, Lico B was found to associated with reduced Th17 cell differentiation and reduce IL-17 production, further attenuate psoriatic inflammation. These findings highlight Lico B as a potential therapeutic agent for psoriasis by concurrently regulating autoimmune and metabolic pathways.

## 2 Methods and materials

### 2.1 Animal experiments

Male C57BL/6 mice, approximately 6–8 weeks old, were purchased from Chongqing Tengxin Experimental Animals Co., Ltd. (Chongqing, China). All animal procedures conform to accordance with the guidelines of the Committee on the Protection and Use of Animals in the Southwest Jiaotong University (SWJTU-2107–004(SWJTU)). After adaptive feeding for 1 week, mice were randomly assigned to four groups, and a psoriasis-like model was induced using imiquimod (IMQ), a Toll-like receptor 7 (TLR7) agonist, as previously described (Qian et al., 2024). In the IMQ group, mice were topically treated with 62.5 mg of commercial 5% IMQ cream (SICHUAN MED-SHINE PHARMACEUTICAL CO., LTD.) daily for six consecutive days (Qian et al., 2024). In the IMQ + Lico B group, mice were orally administered Lico B dissolved in saline (200 μL) 2 h prior to each IMQ application. Animals were further divided into low-dose (40 mg/kg), medium-dose (80 mg/kg), and high-dose (160 mg/kg) treatment groups. The Lico B group received the same dose of Lico B along with topical vaseline. The Control (CTRL) group administered an equivalent volume of saline and topical vaseline.

To evaluate the potential hepatic and renal toxicity of Lico B, C57BL/6 mice were orally administered Lico B dissolved in saline at different doses (200 μL per mouse; low, 40 mg/kg; medium, 80 mg/kg; high, 160 mg/kg) daily. CTRL mice received an equal volume of saline. After six consecutive days of treatment, tissues were collected for further analysis.

### 2.2 Cell culture and drug processing

The keratinocyte cell line HaCaT was obtained from IMMOCELL and cultured in DMEM (Gibco) supplemented with 10% fetal bovine serum (FBS; F101-01, Vazyme) under standard conditions (37 °C, 5% CO_2_). Cells were divided into four groups during the logarithmic growth phase. The IL-17 group was treated with 100 ng/mL IL-17 (Proteintech; HZ-1113); the Lico B group received 9 µM Lico B (MCE; HY-N3388); the IL-17 + Lico B group was co-treated with IL-17 and Lico B; and the control group was treated with PBS. After 24 h of treatment, cells were processed for subsequent experiments.

### 2.3 MTT assay

HaCaT cells were seeded in 96-well plates and allowed to attach for 24 h. When cell confluence reached 60%–70%, they were treated with different concentrations of Lico B for 24 h. At the end of treatment, 10 μL of MTT solution was added to each well, and cells were incubated for an additional 4 h at 37 °C in 5% CO_2_. The supernatant was then carefully removed, and 100 μL of DMSO was added to dissolve the formazan crystals. Absorbance was measured at 560 nm using a microplate reader.

### 2.4 Histology

After fixation, the skin tissue samples from IMQ models were dehydrated through a graded alcohol series, embedded in paraffin, and sectioned into 5 µm slices. The sections were then stained with Hematoxylin-Eosin (Solarbio, G1120) reagents following the manufacturer’s protocol. Pathological alterations in the skin tissues were examined under an optical microscope.

### 2.5 Immunofluorescence

Paraffin-embedded skin tissue sections were deparaffinized in xylene, rehydrated, and subjected to heat-induced antigen retrieval in citrate buffer at 100 °C for 15 min. To prevent nonspecific binding, sections were blocked with 10% goat serum in PBS for 1 h at room temperature. The slides were then incubated overnight at 4 °C with primary antibodies, including rabbit anti-SCD1 (Proteintech, 28678-1-AP, 1:200), rabbit anti-Ki67 (Proteintech, 12348-1-AP, 1:100), and rabbit anti-KRT17 (Proteintech, 17516-1-AP, 1:100). Following primary antibody incubation, sections were washed and incubated with Alexa Fluor 555 goat anti-rabbit IgG (Abcam, ab150078, 1:5,000), Alexa Fluor 488 goat anti-rabbit IgG (Abcam, ab150077, 1:5,000) and/or Alexa Fluor 647 goat anti-rabbit IgG (Abcam, ab150079, 1:5,000) for 2 h at room temperature. Nuclei were counterstained with DAPI staining solution. After washing three times in PBS, coverslips were mounted using 50% glycerol in PBS to prevent photobleaching. Fluorescence images were captured using a laser scanning confocal microscopy (Olympus, FV12-IXCOV).

### 2.6 Preparation of single-cell suspensions and flow cytometry

Single-cell suspensions were prepared according to published methods for flow cytometry [PMID: 32019420]. Spleens were harvested from IMQ models and mechanically dissociated through a 70 μm cell strainer to obtain single-cell suspensions. Red blood cells were lysed using RBC lysis buffer, and the remaining cells were washed and resuspended in PBS for downstream analyses. To evaluate IL-17A expression, single-cell suspensions were stimulated for 4 h at 37 °C using a Cell Activation Cocktail (423,303, Biolegend). After stimulation, surface markers were stained with anti-mouse CD8 antibodies (eBioscience) on ice for 30 min. IL-17 was detected using an anti-mouse IL-17 antibody (eBioscience) following overnight incubation at 4 °C. Samples were analyzed using an LSR Fortessa flow cytometer (SONY, MA900).

### 2.7 Western blot

Total proteins were isolated from the cells with RIPA buffer (Beyotime, P0013B), followed by centrifugation of the lysates at 12,000×g for 30 min at 4 °C. Protein concentration was measured using a BCA assay kit (Solarbio, PC0020). Equal quantities of protein were loaded per lane and resolved on 10% SDS–PAGE gels, separated electrophoretically, and transferred onto PVDF membranes (Millipore, IPVH00010). After blocking, the membranes were probed with primary antibodies against SCD1 (Proteintech, 28678-1-AP, 1:2000), KRT17 (Proteintech, 17516-1-AP, 1:2000) and Beta Actin (Proteintech, 20536-1-AP, 1:10,000) overnight at 4 °C. Following three washes with TBST, the membranes were treated with secondary antibody (Proteintech, SA00001-2, 1:5,000) at room temperature for 2 h and imaged using an eBlot Touch (TOUCH IMAGER PRO). Band intensities were quantified with ImageJ software.

### 2.8 Quantitative real-time PCR

Total RNA was isolated using TRIzol reagent (Vazyme, R411-01) and converted into cDNA using HiScript II Reverse Transcriptase (Vazyme, R223-01) according to the manufacturer’s instructions. Quantitative real-time PCR (qRT-PCR) was conducted on a Real-Time PCR Instrument (Bio-Rad, CFX96 Touch PCR) with 2× TaqSYBR Green qPCR Mix (Vazyme, SQ101). Gene expression levels were quantified using the comparative 2^-ΔΔCT^ method, with GAPDH or β-actin serving as internal reference genes for normalization. The primer sequences of the target and control genes were as follows: *il17* forward:5′CTCAGACTACCTCAACCGTTCC3′ and reverse, 5′ATG​TGG​TGG​TCC​AGC​TTT​CC3′; *rorγt* forward:5′GACCCACACCTCACAAATTGA3′andreverse,5′AGTAGGCCACATTACACTGCT3′; *GAPDH* forward:5′AGGTCGGTGTGAACGGATTTG3′ and reverse, 5′TGT​AGA​CCA​TGT​AGT​TGA​GGT​CA 3′.

### 2.9 Data collection

Targets of Lico B were retrieved from the Traditional Chinese Medicine Systems Pharmacology Database and Analysis Platform (TCMSP). Psoriasis-related gene expression data (GSE54456) were obtained from the NCBI Gene Expression Omnibus (GEO) database. Psoriasis-associated target genes were collected from GeneCards and the Therapeutic Target Database (TTD). The intersection of Lico B targets, psoriasis-related genes from the GSE30528 dataset, and known psoriasis-associated genes was then analyzed to identify potential key targets for subsequent investigation.

### 2.10 Functional enrichment analysis of GO, KEGG

Gene Ontology (GO) and Kyoto Encyclopedia of Genes and Genomes (KEGG) enrichment analyses were performed using the DAVID database for Lico B targets and the targets within the floralwhite module. Enrichment was assessed across biological process, cellular component, and molecular function categories. KEGG pathway enrichment analysis was performed using a significance threshold of P < 0.05, and the top 20 pathways were ranked in descending order based on the calculated p-values.

### 2.11 Identification of psoriasis-associated hub genes via WGCNA

Weighted gene co-expression network analysis was performed using the ‘WGCNA’ R package on differentially expressed genes from the GSE54456 dataset, which included 174 samples. A soft-thresholding power of 19 was selected to construct a scale-free network. The adjacency matrix was then transformed into a topological overlap matrix (TOM), and gene connectivity and phase differences were assessed. Modules were identified using hierarchical clustering combined with the dynamic tree cut method. Gene significance and module membership correlations were calculated, and genes from the relevant modules were extracted for further analysis.

### 2.12 Molecular docking

The crystal structure of SCD1 was obtained from the Protein Data Bank (PDB). Molecular docking of SCD1 with Lico B was performed using AutoDock Tools. The docking results were visualized in three dimensions using PyMOL, and protein–ligand interactions were further illustrated in 2D using Discovery Studio Visualizer.

### 2.13 Statistical analysis

All data are expressed as mean ± standard deviation (S.D.) from at least three independent experiments to ensure reliability. Statistical analysis was performed using R packages or GraphPad Prism. One-way ANOVA was applied for comparisons across multiple groups, and unpaired t-tests were used for two-group comparisons. A P value <0.05 was considered statistically significant.

## 3 Result

### 3.1 Functional enrichment analysis of lico B-associated target genes

**FIGURE 1:**
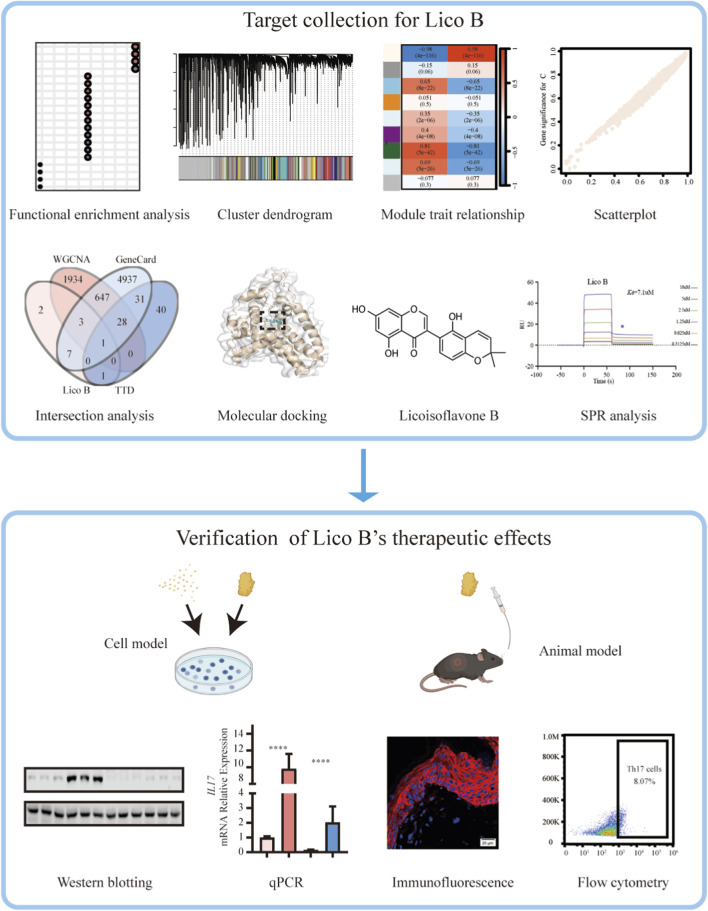
Integrated workflow for target prediction and experimental validation of Lico B in psoriasis. Bioinformatic analyses, including WGCNA module construction, gene–trait correlation analysis, multi-database target intersection (GEO, GeneCards, TTD), molecular docking, and SPR analysis were used to identify candidate targets of Lico B. Subsequent *in vitro* and *in vivo* experiments—comprising Western blotting, qPCR, immunofluorescence staining, and flow cytometry in IL-17–stimulated keratinocytes and IMQ-induced psoriatic mice—were conducted to validate the therapeutic mechanisms of Lico B.

**FIGURE 2:**
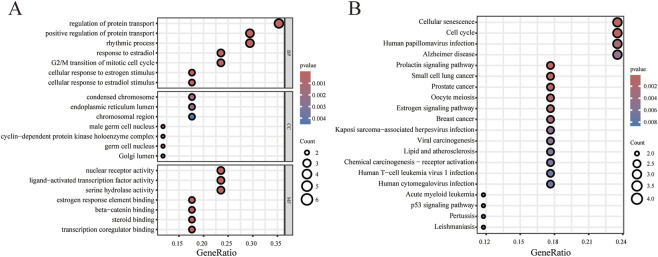
Functional enrichment analysis of Lico B target genes. **(A)** Gene Ontology functional analysis of lico B target genes. **(B)** Kyoto Encyclopedia of Genes and Genomes pathway analysis of lico B target genes.

The workflow of this study was shown in Figure 1. To explore the potential molecular mechanisms of Lico B, we first predicted its targets using TCMSP and subsequently performed Gene Ontology (GO) functional annotation and Kyoto Encyclopedia of Genes and Genomes (KEGG) pathway enrichment analyses. GO analysis revealed that these candidate targets were primarily enriched in biological processes related to estrogen response, cell cycle regulation, and nuclear receptor activity (Figure 2A). KEGG enrichment further indicated significant involvement of these targets in the cell cycle pathway and cancer-related pathways (Figure 2B). Notably, lipid and atherosclerosis pathway was enriched in Lico B′ targets, suggests the potential correlation between Lico B and lipid metabolism. Considering dysregulated lipid metabolism has been increasingly recognized as a contributing factor to chronic inflammatory skin disorders such as psoriasis, we therefore speculate that Lico B may play anti-psoriasis roles via modulation of lipid metabolic processes (Zhang X. et al., 2022; Kanno et al., 2023).

### 3.2 Exploring core psoriasis-related genes using WGCNA analysis

**FIGURE 3:**
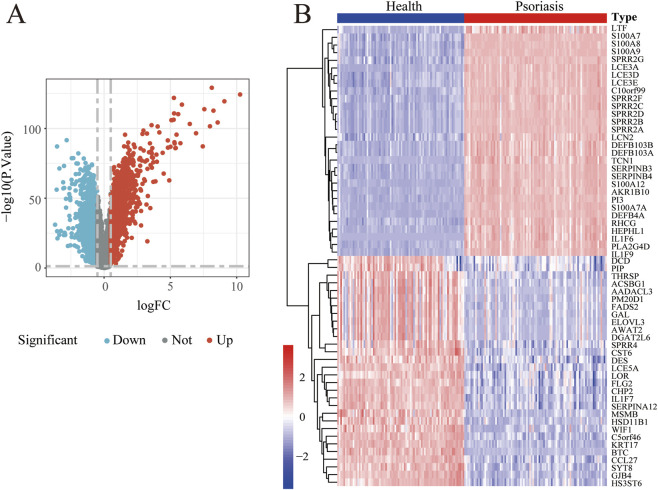
DEGs in psoriasis patients versus healthy controls. **(A)** Volcanic map for DEGs analysis of GSE54456. **(B)** Heat map for DEGs analysis of GSE54456. Blue represents downregulated genes, red represents upregulated genes.

**FIGURE 4:**
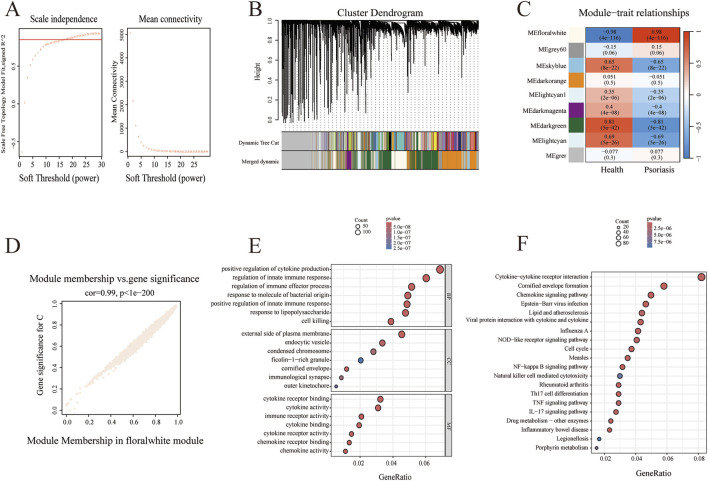
Exploring core psoriasis-related genes using WGCNA analysis. **(A)** Analysis of soft-thresholding power and mean connectivity to determine the scale-free topology fit index of the network. **(B)** The hierarchical clustering of all genes in GSE54456 dataset. **(C)** Module-trait heatmap of the correlation between the clustering gene module and psoriasis in GSE54456. **(D)** Scatter plot of module floralwhite has the strongest positive correlation with psoriasis. **(E)** The GO enrichment results visualized in a bubble plot. **(F)** KEGG enrichment results displayed as a bubble plot.

To further identify potential molecular targets associated with psoriasis, we analyzed the GSE54456 dataset from the GEO database, which includes 92 psoriatic lesions and 82 healthy skin biopsy samples. Differential expression genes (DEGs) analysis was performed, and the volcano plot and heatmap demonstrated distinct sets of upregulated and downregulated genes between psoriatic and healthy tissues (Figures 3A,B). To further characterize disease-related transcriptional patterns, WGCNA was performed. According to the WGCNA principle, an appropriate soft-thresholding power (β) is required to construct an adjacency matrix that satisfies the scale-free topology criterion. Candidate power values ranging from 1 to 30 were evaluated, and for each value, the scale-free topology fit index and mean connectivity were calculated (Figure 4A). The left panel illustrates the relationship between different power values and the scale-free topology fit, whereas the right panel depicts the corresponding mean connectivity of the constructed networks. These analyses indicated that a soft-thresholding power of β = 19 achieved an optimal balance between high scale-free topology fit and adequate mean connectivity. Therefore, β = 19 was selected for subsequent network construction. Using this selected power, a WGCNA was established, resulting in the identification of 9 distinct gene modules. These modules were visualized using a hierarchical clustering dendrogram (Figure 4B), in which the upper panel represents gene clustering based on topological overlap, and the lower panel indicates module assignment. Genes sharing the same color were classified into the same module, while the colors labeled as “Dynamic Tree Cut” indicate modules initially identified using the dynamicTreeCut algorithm. Given the high similarity among certain modules, closely related modules were further merged, as shown in the “Merged dynamic” annotation. A heatmap depicting the clustering of all genes was also generated (Figure 4C). Module–trait associations were subsequently assessed by calculating Pearson correlation coefficients and corresponding p values between module eigengenes and clinical traits. As shown in Figure 4D, the floralwhite module, comprising 2,617 genes, exhibited the strongest positive correlation with psoriasis (cor = 0.98, p = 4 × 10^−116^). Consequently, the floralwhite module was selected for further analysis. A scatter plot revealed a strong positive correlation between gene significance (GS) and module membership (MM) within the floralwhite module (cor = 0.99, p = 1 × 10^−200^), indicating that genes highly associated with psoriasis were also central components of this module. Next, we conducted an in-depth study of gene functions in the floralwhite module. GO functional enrichment analyses indicated that the enriched biological processes were mainly associated with positive regulation of cytokine production, regulation of innate immune response and regulation of immune effector process. In terms of cellular components, the targets were related to predominantly enriched external side of plasma membrane, cornified envelope and endocytic vesicle. Regarding molecular function, the targets were predominantly enriched in cytokine and chemokine-related activities (Figure 4E). Subsequent KEGG pathway analysis of the floralwhite module indicated that the potential pathways including cytokine–cytokine receptor interaction, cornified envelope formation, chemokine signaling pathway, lipid and atherosclerosis and Th17 cell differentiation (Figure 4F). These findings suggested that psoriasis involves immune dysregulation, imbalanced proliferation and differentiation of keratinocytes, and accompanying lipid metabolism disorders.

### 3.3 SCD1 is a potential therapeutic target of lico B in psoriasis

**FIGURE 5:**
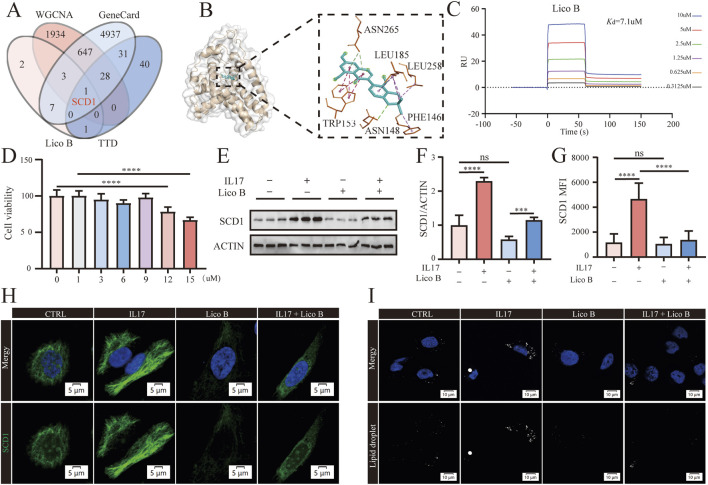
SCD1 is a potential therapeutic target of Lico B in psoriasis. **(A)** Venn diagram showing SCD1 as the overlapping therapeutic target. **(B)** The molecular docking results of lico B and SCD1. **(C)** SPR analysis of the binding affinity of Lico B to full-length SCD1 protein. **(D)** Cell viability of lico B treated HaCat cells. **(E)** and **(F)** Western blot analysis of SCD1 levels in different groups. **(G)** and **(H)** Immunofluorescence staining and analysis of SCD1. **(I)** Representative immunofluorescence images of lipid droplet staining. All data presented in this study are representative of at least three independent experiments, and the results are expressed as means ± standard deviation (s.d.).

To identify key targets of Lico B in psoriasis, we integrated multiple datasets for intersection analysis. Specifically, we compared the predicted targets of Lico B, the psoriasis-related gene set from the floralwhite module, and psoriasis-associated genes from the Therapeutic Target Database and GeneCards Databases were analyzed. The integrated analysis yielded a single overlapping gene SCD1. This indicates that SCD1 is both a core regulatory gene in psoriatic pathology and a potential direct target of Lico B intervention (Figure 5A). Molecular docking revealed favorable binding of Lico B within the SCD1, involving tryptophan, asparagine, leucine and phenylalaninem, with a calculated binding energy of −11.7 kcal/mol (Figure 5B). Surface plasmon resonance assays (SPR) further confirmed that Lico B directly interacted with SCD1 protein in a positive dose-dependent manner. The determined equilibrium dissociation constant (Kd) for Lico B binding to the SCD1 protein was 7.1 μmol/L (Figure 5C). Given that keratinocytes are key effector cells in psoriasis, we next examined the effect of Lico B in IL-17A treated HaCaT cells. Cell viability assays confirmed that Lico B exhibited no detectable cytotoxicity at concentrations ≤12 μM after 24 h (Figure 5D). 9 μM was therefore selected for subsequent experiments. Western blot analysis showed that IL-17A stimulation markedly increased SCD1 expression in keratinocytes, whereas Lico B treatment effectively suppressed this upregulation (Figures 5E,F). Consistently, immunofluorescence staining revealed significantly reduced SCD1 expression in the Lico B+ IL17 group compared to the IL17 group *in vivo* (Figures 5G,H). Because SCD1 regulates the synthesis of monounsaturated fatty acids and thereby influences neutral lipid storage (Su et al., 2023; Sarandi et al., 2023; Kanno et al., 2023). We further examined intracellular lipid droplets as a downstream readout of SCD1 linked lipid metabolism in HaCaT cells. A downstream production of SCD1, intracellular lipid droplet was assessed in HaCaT cells. Immunofluorescence staining of lipid droplet showed that IL-17A stimulation increased lipid droplet accumulation, whereas Lico B treatment markedly reduced this accumulation (Figure 5I), indicating that Lico B may modulate lipid metabolism by targeting SCD1.

### 3.4 Lico B attenuates IL17A-induced the proliferation of HaCaT cells

**FIGURE 6:**
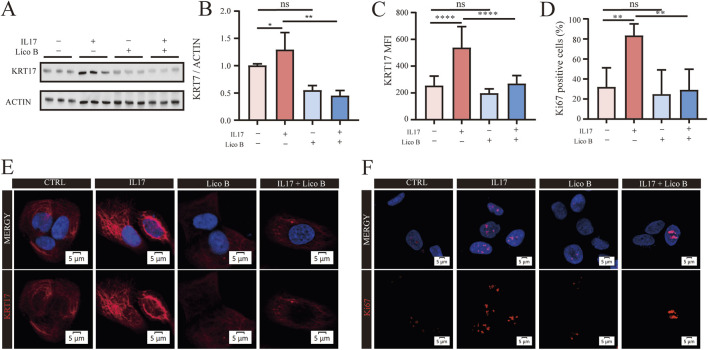
Lico B attenuates IL17A-induced proliferation in HaCaT Cells. **(A)** and **(B)** Western blot analysis of KRT17 levels in different groups. **(C–F)** Representative immunofluorescence images and analysis of KRT17 and Ki67 in different groups. All data presented in this study are representative of at least three independent experiments, and the results are expressed as means ± standard deviation (s.d.).

Dysregulated keratinocyte proliferation is a fundamental driver of epidermal hyperplasia in psoriasis. Pathway enrichment analysis indicated that Lico B associated targets are enriched in cornified envelope formation and cell cycle regulation in Figure 2, suggesting Lico B in the control of keratinocyte differentiation and proliferation. We therefore examined whether Lico B could suppress IL-17–induced proliferative responses in HaCaT cells. Western blot analysis showed that co-treatment with IL-17 and Lico B reduced the expression of the hyperproliferation marker KRT17 compared with IL-17 alone (Figures 6A,B). Consistently, immunofluorescence staining demonstrat significantly decreased expression of both KRT17 and the proliferation marker Ki67 in the Lico B + IL17 group compared with the IL-17–stimulated group (Figures 6C–F). These findings indicate that Lico B effectively attenuates IL-17-driven keratinocyte hyperproliferation, suggesting its potential to ameliorate psoriatic epidermal pathology.

### 3.5 Lico B ameliorates psoriatic phenotypes in an IMQ-induced psoriasis-like mouse model

**FIGURE 7:**
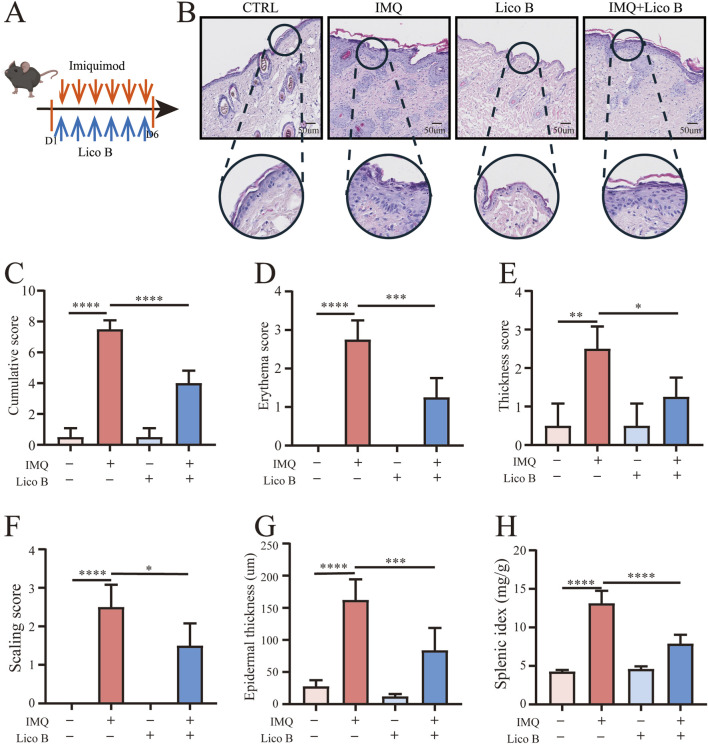
Lico B ameliorates psoriatic phenotypes in an IMQ-induced psoriasis-like mouse model. **(A)** Experimental scheme of the imiquimod (IMQ)-induced psoriasis-like mouse model with lico B. **(B)** Representative histologic sections of dorsal skin from control, IMQ, Lico B and IMQ + Lico B–treated mice. **(C–F)** The Psoriasis Area and Severity Index (PASI) scores of the skin tissue. **(G)** The average epidermal thickness. **(H)** Spleen weight index. All data presented in this study are representative of at least three independent experiments, and the results are expressed as means ± standard deviation (s.d.).

To assess the therapeutic effects of Lico B, we established a psoriasis-like mouse model induced by the TLR7 agonist IMQ (Figure 7A). On the day of sacrifice, we observed that mice in the IMQ group exhibited pronounced erythema, increased scaling, and marked epidermal thickening. Treatment with Lico B alleviated these symptoms, leading to reduced erythema, scaling, and skin thickness. The PASI scores were significantly reduced in the Lico B-treated IMQ model group compared with the IMQ group (Figures 7C–F). To further assess the histopathological effects of Lico B, hematoxylin and eosin (H&E) staining of back skin sections was performed. Results showed that the IMQ group displayed pronounced epidermal hyperkeratosis, acanthosis, elongation of rete ridges, and increased infiltration of immune cells in the dermis. In contrast, these psoriatic pathological features were markedly alleviated in the IMQ + Lico B group (Figure 7B). Moreover, we found that Lico B treatment significantly reduced epidermal thickness and decreased spleen size compared with the IMQ group (Figures 6G,H). Collectively, these findings indicate that Lico B effectively mitigates IMQ-induced psoriasis-like skin inflammation and epidermal hyperplasia.

### 3.6 Lico B ameliorates epidermal hyperproliferation and immune dysregulation in a psoriatic mouse model

**FIGURE 8:**
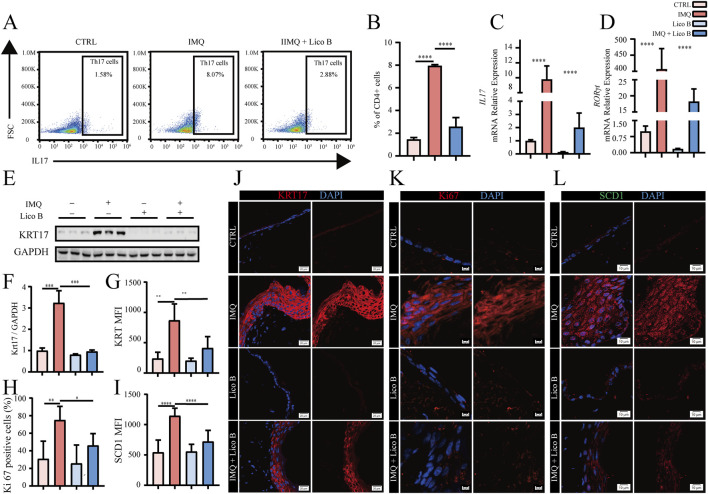
Lico B amelioratesattenuates epidermal hyperproliferation and immune dysregulation in a psoriatic mouse model. **(A)** and **(B)** Representative flow cytometry diagrams and statistical analysis of splenic Th17 cells in the different mouse model groups. **(C)** and **(D)** RT-qPCR analysis of *IL17 and RORγt* mRNA expression levels in each mouse model group. **(E)** and **(F)** Western blot analysis of KRT17 levels in different groups. **(G–L)** Immunofluorescence staining and analysis of KRT17, Ki67 and SCD1. All data presented in this study are representative of at least three independent experiments, and the results are expressed as means ± standard deviation (s.d.).

Th17 cell differentiation and its effector cytokine IL-17 are central drivers of psoriatic phenotypes (Blauvelt and Chiricozzi, 2018; Li et al., 2020; Shen et al., 2024). Therefore, we next examined whether Lico B could modulate Th17 responses in IMQ-induced psoriasis-like mice. Flow cytometry analysis revealed that the proportion of splenic Th17 cells in the spleen was significantly reduced in the IMQ + Lico B group compared with the IMQ model group (Figures 8A,B). Consistently, qRT-PCR analysis of dorsal skin demonstrated that Lico B treatment markedly decreased the expression of *IL-17* and its key transcriptional factor *RORγt* mRNA expression (Figures 8C,D), suggesting that Lico B suppresses Th17 cell differentiation and IL-17 production. To evaluate the impact of Lico B on keratinocyte proliferation *in vivo*, we next assessed the expression of hyperproliferation markers KRT17 and Ki67 in skin tissues. Western blot results showed that KRT17 protein levels were significantly reduced in the Lico B + IMQ group compared with the IMQ group (Figures 8E,F). Immunofluorescence staining further confirmed that Lico B treatment significantly decreased KRT17 and Ki67 expression in psoriatic mouse skin tissue (Figures 8G,H,J,K). Because SCD1 is a key lipid metabolic enzyme upregulated in our psoriatic model, we also examined its expressions in skin lesions. Immunofluorescence staining revealed that SCD1 level was also reduced in the epidermis of IMO-induced mice following Lico B administration (Figures 8I,L). Given that IL-17A promotes keratinocyte hyperproliferation through induction of IL-19 and IL-36γ (Nograles et al., 2008; Sa et al., 2007), the coordinated reduction in Th17 differentiation, IL-17 expression and epidermal hyperproliferation suggests that Lico B alleviates epidermal hyperproliferation in psoriasis-like mice by inhibiting Th17 cell differentiation and attenuating IL-17 signaling.

## 4 Discussion

Licorice is a commonly used medicinal plant with multiple health benefits. Beyond its applications as a flavoring agent, food additive, and dietary supplement, it has been employed for expectorant purposes and the treatment of allergic asthma (Ding et al., 2022). Lico B, one of the bioactive constituents of licorice, has been extensively investigated in the context of infectious diseases (Faisal Ahmed et al., 2025; Liu et al., 2019; Akiyama et al., 2010). Psoriasis, a chronic inflammatory skin disorder, has recently attracted growing interest regarding treatment with traditional Chinese medicines. Given the potent anti-inflammatory effect of Lico B, its potential therapeutic value in psoriasis has become an emerging area of interest. Our findings has for the first time demonstrated the therapeutic potential of Lico B in psoriasis and elucidate its underlying molecular mechanisms. Lico B markedly ameliorates psoriatic lesions by modulating inflammatory immune responses and suppressing excessive keratinocyte proliferation.

Psoriasis is a chronic inflammatory skin disorder primarily driven by immune dysregulation, in which the activation of Th17 cells and the secretion of their associated cytokines play a pivotal role in disease development. In genetically susceptible individuals, environmental triggers activate dendritic cells, which subsequently release IL-23 to stimulate Th cell populations and promote their differentiation into Th17 cells. Th17 effector cytokines, including IL-17A, IL-17F, IL-21, IL-22, and TNF-α, further activate keratinocytes, leading to the production of chemokines, antimicrobial peptides, and enhanced proliferation, thereby sustaining a pathogenic inflammatory loop (Li et al., 2020; Sieminska et al., 2024). In our study, we observed that Lico B treatment reduced the proportion of Th17 cells in psoriasis-like mice and markedly decreased the expression of *IL17A* and the Th17 differentiation transcriptional factor *RORγt*, indicating that Lico B has the potential to modulate immune responses in psoriasis.

Keratinocytes serve as the primary target cells for IL-17A, with constitutive expression of IL-17A receptors throughout the epidermal layer (Nograles et al., 2008; Harper et al., 2009; Chiricozzi et al., 2011). Aberrant IL-17A expression and activation of the IL-17 signaling pathway significantly contribute to psoriasis progression (Chiricozzi et al., 2011; Krueger et al., 2012). IL-17A stimulates keratinocyte proliferation with the assistance of IL-19 and IL-36γ (Lowes et al., 2014; Shi et al., 2011). Multiple clinical studies have shown that blocking IL-17A or its receptor can reverse hyperkeratosis and scaling in approximately 80% of psoriasis patients (Leonardi et al., 2012; Krueger et al., 2012; Yiu et al., 2022), underscoring the link between IL-17 signaling and epidermal proliferation. Consistently, our results demonstrate that Lico B inhibits the expression of proliferation markers KRT17 and Ki67 in psoriasis-like mouse skin and attenuates IL-17A–induced upregulation of KRT17 and Ki67 in keratinocytes. These findings indicate that Lico B not only involved in regulating Th17 cell differentiation but also suppresses keratinocyte hyperproliferation by interfering with the IL-17 signaling pathway.

Metabolic imbalance, particularly in lipid metabolism, has emerged as an important contributor to the initiation and progression of psoriasis. Numerous studies have shown that psoriatic epidermis exhibits abnormal expression of lipid-metabolizing enzymes accompanied by significant disruptions in lipid composition and homeostasis (Li et al., 2025; Owczarczyk-Saczonek et al., 2020). By integrating target prediction, differential gene expression analysis, WGCNA and molecular docking, we identified SCD1—the rate-limiting enzyme in fatty acid desaturation—as a core node linking of Lico B to psoriasis pathophysiology. In our vivo and *in vitro* model, SCD1 was markedly upregulated in psoriatic skin and in IL-17–stimulated keratinocytes, wheras Lico B treatment effectively reduced SCD1 expression and was accompanied by decreased intracellular lipid droplet accumulation, indicating a partial normalization of lipid handing under psoriatic conditions.

SCD1 catalyzes the desaturation of saturated fatty acids (SFAs) to generate monounsaturated fatty acids (MUFAs), predominantly oleic acid (C18:1) and palmitoleic acid (C16:1). These MUFAs serve as essential building blocks of triglycerides, wax esters, and cholesterol esters and are crucial for maintaining membrane composition, endoplasmic reticulum homeostasis, and cellular energy storage. Aberrant SCD1 activity disrupts this desaturation process and leads to lipid metabolic imbalance and excessive lipid accumulation—pathological features that have been linked to multiple inflammatory diseases (Liu et al., 2020; Flori et al., 2023; Bogie et al., 2020). Although studies in cancer biology have demonstrated that SCD1-driven remodeling promotes lipid storage and enhances the proliferative and regenerative capacity of cells (Li et al., 2009; Zang et al., 2025), its functional contribution to psoriatic skin inflammation and epidermal hyperplasia remained unclear. Our findings begin to address this gap by showing that SCD1 and its downstream readout—lipid droplets—display expression patterns paralleling those of the proliferation markers KRT17 and Ki67 in both keratinocyte cultures and IMQ-induced psoriatic lesions. These correlative data, together with the ability of Lico B to simultaneously downregulate SCD1, reduce lipid droplet accumulation, and limit keratinocyte hyperproliferation, support a model in which excessive SCD1 activity contributes to epidermal thickening in psoriasis by disturbing lipid homeostasis and creating a metabolic environment permissive for sustained proliferation. However, we did not directly manipulate SCD1 activity in this study, and future genetic or pharmacologic gain- and loss-of-function experiments will be required to formally establish causality and to define the specific lipid species and signaling pathways involved. Within these limitations, our results suggest that targeting SCD1-dependent lipid metabolic reprogramming represents a promising strategy to restore epidermal metabolic–immune balance in psoriasis and may complement existing therapies directed at the Th17/IL-17 axis.

**FIGURE 9:**
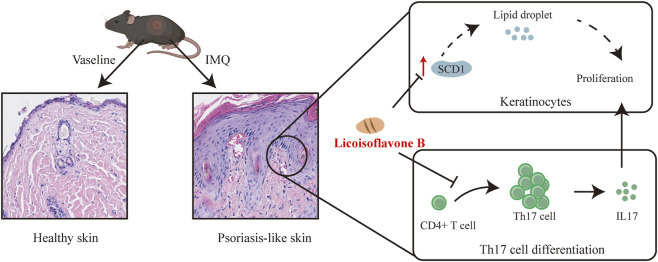
Schematic illustration of lico B treatment in psoriasis. Lico B modulates lipid metabolic pathways by suppressing SCD1-dependent metabolic reprogramming and reducing lipid droplet accumulation in keratinocyte, while simultaneously attenuating the TH17/IL-17 axis. Through concurrent regulation of keratinocyte metabolism and inflammatory cytokine production, Lico B ameliorate psoriatic skin pathology.

In summary, this study reveals, for the first time, the molecular mechanisms underlying Lico B’s therapeutic effects in psoriasis. Lico B modulates immune function, leading to reduced expression of the pathogenic cytokine IL-17A. Additionally, Lico B may alleviate epidermal lesions by improving dysregulated lipid metabolism through targeting SCD1. Collectively, our findings demonstrate that Lico B controls psoriasis-associated immune responses and restores lipid homeostasis, representing a promising therapeutic strategy for psoriasis (Figure 9).

## Data Availability

The datasets presented in this study can be found in online repositories. The names of the repository/repositories and accession number(s) can be found below: https://www.ncbi.nlm.nih.gov/, GSE54456.

## Supplementary material

SUPPLEMENTARY FIGURE S1 Effects of Lico B at different doses on IMQ-induced psoriasis-like mice. **(A)** Representative histologic sections of skin from control, IMQ, IMQ + low-dose Lico B (IMQ+Low), IMQ + medium-dose Lico B (IMQ+M), and IMQ + high-dose Lico B (IMQ+High). **(B,C)** The Psoriasis Area and Severity Index (PASI) scores of the skin tissue. **(D)** The average epidermal thickness. **(E,F)** Serum levels of ALT and AST in different doses of Lico B group. **(H)** Serum BUN levels in different doses of Lico B group. **(G,J)** Representative H&E-stained liver sections and quantification of necrotic areas from mice treated with different doses of Lico B. **(I,K)** Representative H&E-stained kidney sections and tubular injury scores from mice treated with different doses of Lico B. All data presented in this study are representative of at least three independent experiments, and the results are expressed as means ± standard deviation (s.d).
