# Supplementary figures and images for: Licoisoflavone B alleviates psoriasis via SCD1-targeted lipid metabolism reprogramming and suppression of Th17/IL-17–mediated inflammation
Source: Front Pharmacol. 2026 Feb 16;17:1754729. doi: 10.3389/fphar.2026.1754729 (PMC12951635; doi:10.3389/fphar.2026.1754729)

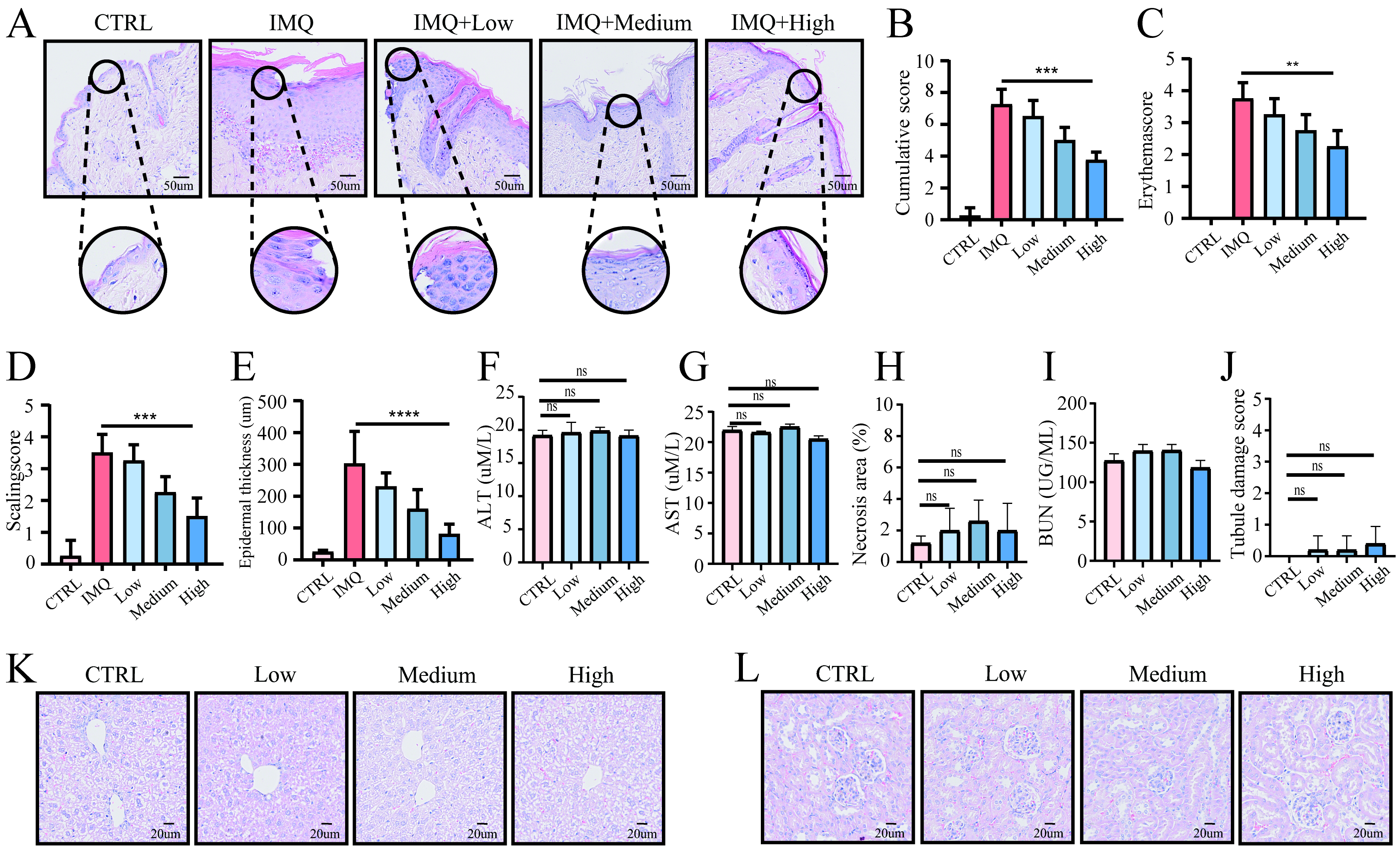

Supplement: Supplementary file 1 [file Image1.tif]
